# Concurrent validation of OpenCap for identifying ACL re-injury risk factors during a drop jump test in a healthy cohort

**DOI:** 10.1038/s41598-026-44758-0

**Published:** 2026-03-24

**Authors:** Bernhard Färber, Brian Horsak, Florian Kurt Paternoster

**Affiliations:** 1 TUM School of Medicine and Health, Associate Professorship of Biomechanics in sport, Technical University of Munich, Am Olympiacampus 11, 80809 Germany, Munich, Germany; 2 Center for Digital Health and Social Innovation, St. Pölten University of Applied Sciences, Campus-Platz 1, St. Pölten, 3100 Austria; 3 Institute of Health Sciences, St. Pölten University of Applied Sciences, Campus- Platz 1, St. Pölten, 3100 Austria

**Keywords:** Anterior cruciate ligament injuries, Markerless motion capture, 3D kinematic analysis, Biomechanical validation, Re-injury prevention, Three-dimensional imaging, Musculoskeletal system

## Abstract

3D motion analysis (3DMA) can help identify patients at increased risk of ACL re-injury, but traditional marker-based systems have limited clinical accessibility. OpenCap, a novel, low-cost, markerless system, aims to enhance accessibility to 3DMA. This study evaluated the concurrent validity of a modified OpenCap version using a 2-DOF knee model for kinematics, while kinetics and ground reaction forces were derived using the native 1-DOF model, compared to a marker-based system. Twenty-four healthy participants performed 240 drop jumps, with data simultaneously captured by both systems. Root mean square error (RMSE), mean absolute error (MAE), maximum error, Pearson correlation, Bland-Altman plots, and statistical parametric mapping (SPM) were used to analyze inter-system differences. RMSE exceeded 6° for frontal-plane knee kinematics with strong waveform correlations (*r* > 0.90). Transverse-plane hip moments showed normalized MAE < 1% with weak to strong negative correlations. Sagittal-plane knee moments had normalized MAE of 5.6% and strong correlations (*r* > 0.90). Vertical GRFs showed normalized MAE > 6% and strong correlations (*r* > 0.90). SPM identified significant differences across most ground contact phases, and Bland-Altman analyses showed wide agreement limits for knee moment asymmetry at initial contact. OpenCap currently cannot be recommended for ACL re-injury risk assessment but demonstrated potential for increasing 3DMA accessibility.

**Supplementary Information:**

The online version contains supplementary material available at 10.1038/s41598-026-44758-0.

## Introduction

Anterior cruciate ligament (ACL) ruptures are severe injuries that compromise knee stability and proprioceptive function^1,2^. They occur frequently, with more than 40,000 ACL reconstruction surgeries performed annually in Germany and an incidence of 68.6 per 100,000 person-years reported in the United States^3,4^. Rehabilitation is typically guided by time since injury or surgery in combination with clinical and functional criteria, with athletes progressing stepwise from return to training to return to competition^3,5^. Functional performance tests are commonly used to determine readiness for progression and to identify individuals at increased risk of ipsilateral or contralateral ACL re-injury^3,6–8^. Nevertheless, re-injury rates remain high (1.5–37.5%), indicating the need for more sensitive and comprehensive assessment strategies^6^.

Three-dimensional motion analysis (3DMA) is a key tool for studying kinematics and kinetics, with marker-based (MB) systems often referenced as the criterion method. They use reflective markers placed on the body to track movement with infrared cameras^9–12^. Using inverse kinematics or in combination with force plates and inverse dynamic algorithms, precise analyses of joint kinematics and joint moments are possible. However, MB systems are expensive and require a lot of time and expertise, which limits their use in regular clinical settings^13,14^. This complicates the widespread use of MB systems in rehabilitation screenings outside specialized clinics and academic institutions.

New markerless motion analysis systems have been developed using multiple synchronized digital video cameras and advanced machine learning-driven pose estimation algorithms to identify anatomical features and process data^10,12^. These systems do not require reflective markers, making them easier and quicker to use (e.g., no subject preparation time). Although these markerless systems show promising accuracy compared to MB systems for tasks like walking and jumping^15–17^, they remain expensive, still restricting their widespread usage, e.g., in clinical settings.

OpenCap is a new, open-source, smartphone-based 3D markerless motion analysis system that offers an affordable alternative using 2–8 smartphones, a computer, and cloud computing^14^. Its kinematic accuracy has improved across versions, with RMSE reaching 4.1° in v0.3^18^. Independent studies report RMSEs of 5.8° for gait (v0.1)^19–21^, 5.1–18.3° for dynamic movements (unspecified version)^22^, and 1.91–5.41° for return-to-sport tasks (v0.2)^23^. OpenCap can also estimate joint moments and ground reaction forces via muscle-driven simulations without force plates^14^. Developer-reported accuracy shows RMSEs of 1.56% BW×height for joint moments and 6.2% BW for 3D GRFs^14^.

Conducting motion analysis in a laboratory equipped with infrared cameras and force plates provides comprehensive data for assessing ACL reconstruction (ACLR)–related injury risk, but is associated with high costs, complex setup, and the need for technical expertise. While several studies have examined the concurrent validity of markerless systems such as OpenCap against traditional marker-based systems, these investigations have predominantly focused on kinematic outcomes^19,21–23^. However, a comprehensive ACLR screening requires both kinematic and kinetic parameters, including joint moments and ground reaction forces, which are essential for evaluating dynamic knee stability and re-injury risk. The novelty of the present study lies in replicating a clinically relevant, full ACLR motion analysis—encompassing both kinematic and kinetic data—using only smartphone-based recordings with OpenCap.

When evaluating the validity of markerless motion capture for clinical applications, it is essential to consider established accuracy thresholds. For kinematic measures, differences of approximately 2.5°–5° in frontal plane knee motion have been reported as the minimum detectable change during dynamic tasks and have been shown to distinguish between athletes with and without subsequent ACL injuries^24–26^. For kinetic measures, an MAE of ≤ 2.5% of bodyweight-normalized joint moments is accepted as a threshold for sufficient agreement between measurement systems^27^. For ground reaction forces, errors exceeding 10–15% may limit clinical applicability at the individual level. These benchmarks serve as a reference for evaluating the accuracy of the novel OpenCap system compared to a traditional marker-based system in the context of a comprehensive ACL re-injury risk screening encompassing both kinematic and kinetic data.

Hence, this study aimed to evaluate the concurrent validity of OpenCap vs. a traditional MB system in measuring key parameters for ACL reconstruction (ACLR) patients based on published literature^28–30^. These parameters include frontal knee joint kinematics, sagittal knee and transverse hip moments, and vertical ground reaction forces during drop jumps. We aimed to explore whether OpenCap’s results might offer insights relevant to ACL re-injury risk assessment.

## Materials and methods

### Sample size estimation

A priori sample size was estimated using G*Power version 3.1.9.7^31^. Based on existing research on OpenCap^21–23,32–34^, the assumed effect size was d = 0.6. To detect this effect with a two-tailed, paired-samples t-test at an alpha level of 5% and with 80% power, a sample size of 24 participants was required.

### Participants

In total, 24 (17 male, 7 female) participants were recruited (Table 1). All participants were regularly physically active (> 2 times/week) and free of injuries to the lower limbs within the last six months. The study was approved by the local Ethics Committee of the Technical University of Munich (2023-481-S-SB) and conducted according to the Declaration of Helsinki. Participants voluntarily participated and gave written informed consent.

**Table 1:** Sex distribution (n, %) and Participant characteristics (Mean ± SD).

Sex (Male | Female)	17 (71%) | 7 (29%)
Age [years]	26.5 ± 2.7
Height [m]	1.8 ± 0.1
Body mass [kg]	75.0 ± 11.7
BMI [kg/m^2^]	23.9 ± 2.4

### Experimental setup

For marker-based analyses, 15 cameras (Vicon Motion Systems Ltd, Oxford, UK) were used at a sample rate of 240 Hz to record lower-body kinematics using the conventional gait model 2.3 marker set^35^. GRFs were recorded with two force plates (Advanced Mechanical Technology Inc., Watertown, USA) at 1200 Hz. Data was synchronously captured using Vicon Nexus software (version: 2.12.1).

The OpenCap setup consisted of four iOS devices (one *iPhone 13 Pro*, one *iPhone 11 Pro*, two 4th generation *iPad Air;* Apple Inc., Cupertino, USA) using a sample rate of 240 Hz.

**Fig. 1:**
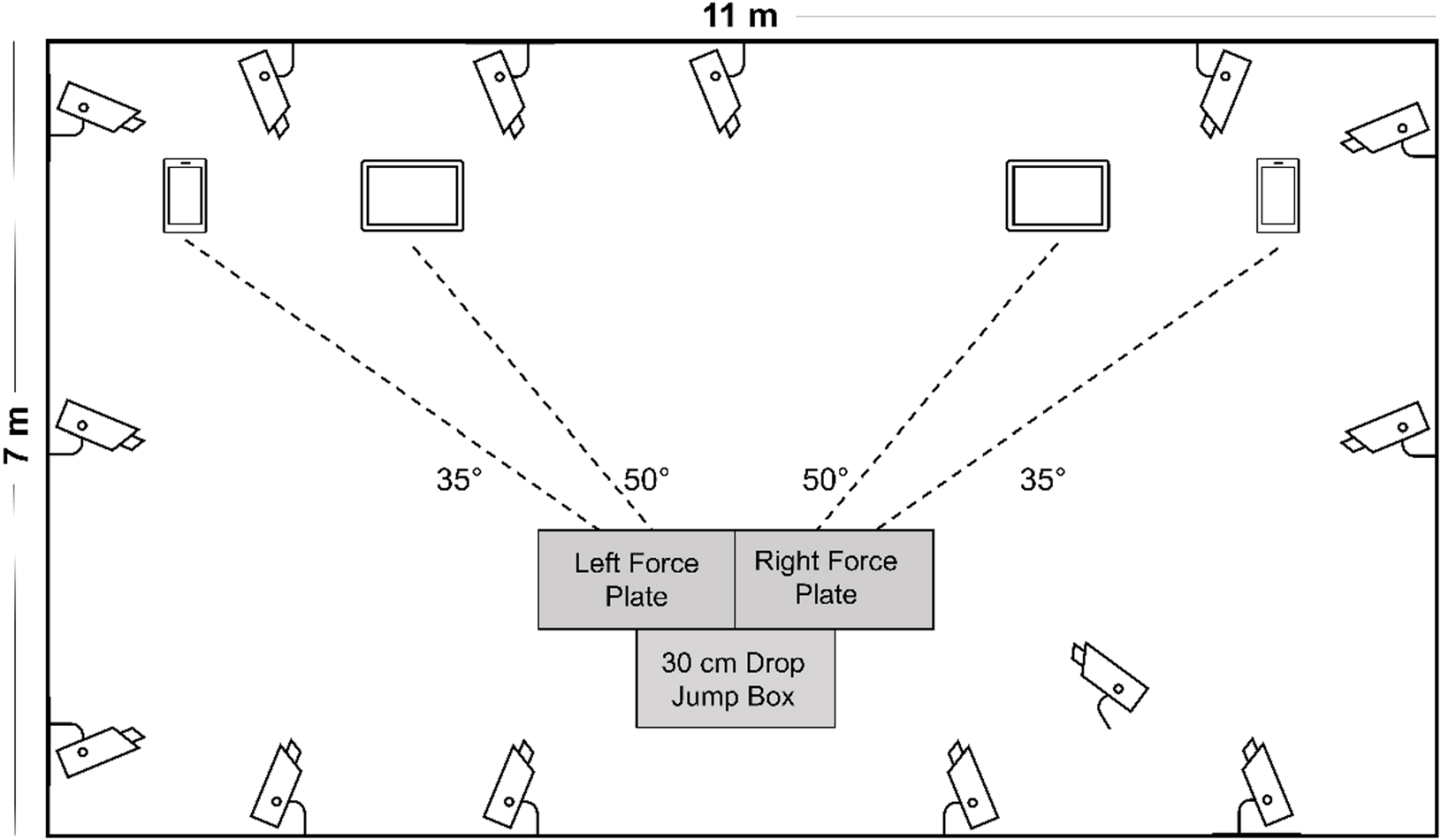
Schematic overview of the laboratory setup. The angles are horizontal angles relative to the force plates. Distance between force plates: 3 mm. The drop jump box was placed immediately adjacent to the force plates. m = meters. cm = centimeters.

The devices were mounted on tripods at a height of 1.5 m, positioned 3 m from the force plates at different angles, and calibrated following OpenCap’s best practice guidelines (Fig. 1).

### Experimental protocol

Each participant completed a single testing session lasting 60–90 min, wearing minimal clothing (all barefoot, dark sports tights for men, additional dark sports bra for women), and performed a warm-up routine of five minutes of stationary cycling at ≥ 50 W and five minutes of dynamic lower limb movements. Participants performed three to five familiarization drop-jumps. They stood on a 30 cm high box, hands on hips, and performed an initialization movement before each drop jump (see Sect. 2.6, “Data analysis”). They were instructed to land with each foot on a separate force plate, minimize ground contact time, and jump as high as possible. After familiarization, participants performed drop jumps, which were recorded by both systems simultaneously, with a one-minute rest between each repetition, until ten valid recordings were obtained concurrently with both systems.

### Data processing

Initial processing occurred in OpenCap’s web application immediately after data capture, where we selected the OpenPose 2D pose-estimation algorithm in combination with OpenCap’s marker augmenter v0.3 to generate kinematic analyses.

**Fig. 2:**
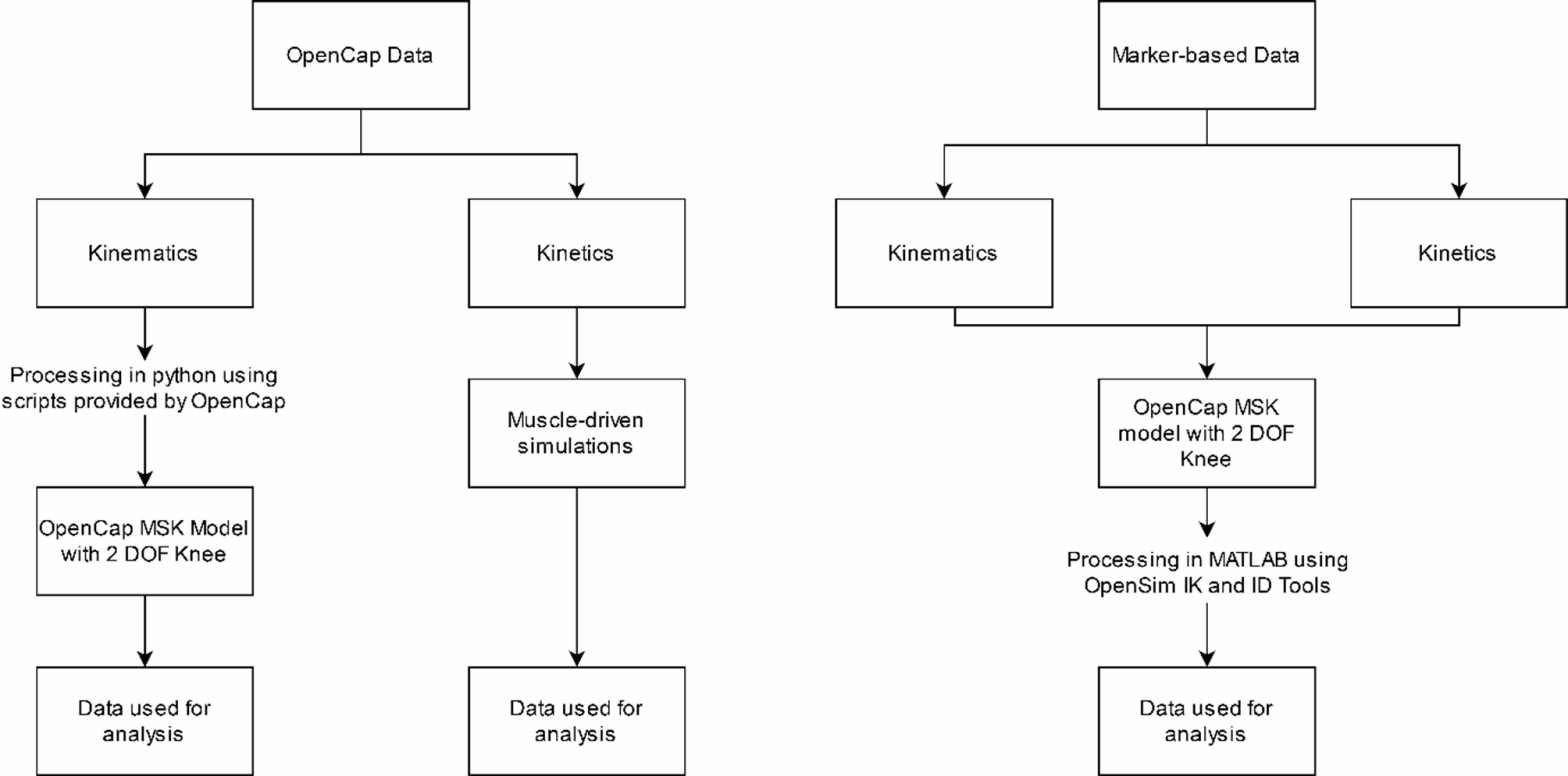
Data pre-processing steps for both motion analysis systems. DOF = Degree(s) of freedom, ID = inverse dynamics, IK = inverse kinematics.

To enable kinematic analyses of frontal plane knee motion, a degree of freedom (DOF) not natively supported by OpenCap, all kinematic data from OpenCap was reprocessed locally using OpenPose on 1 × 736 resolution settings. Open-source code provided by OpenCap’s developers on GitHub (https://github.com/stanfordnmbl/opencap-core) was used to modify OpenCap’s default musculoskeletal model (MSK)^36^ to allow for frontal plane knee motion analyses. In contrast, no changes to the native MSK used by OpenCap were necessary for estimating kinetic data via dynamic, muscle-driven simulations. The simulation time window was set to 200 ms before initial contact (IC) and until 200 ms after lift-off (LO), as determined by the force plates (threshold 20 N). No changes were made to the default muscle-driven simulation settings (for further details see: https://github.com/stanfordnmbl/opencap-processing/tree/main/UtilsDynamicSimulations/OpenSimAD). The three-dimensional marker trajectories obtained from the MB motion capture system were used to anthropometrically scale a generic musculoskeletal model within OpenSim 4.0. Following model scaling, inverse kinematics and inverse dynamics analyses were performed using OpenSim’s^37^ dedicated toolchain to estimate joint kinematics and joint moments, respectively. To maintain methodological alignment across conditions, both systems were processed using the 2-DOF knee musculoskeletal model for the kinematic analyses. For kinetic analyses, however, the marker-based data were processed using the same 2-DOF model, whereas OpenCap kinetics were computed using its native 1-DOF knee model due to current software constraints^36,38,39^. Data was processed using Matlab (R2021b, MathWorks, Natick, USA) and scripts based on the code available from^40^. Figure 2 gives an overview of the processing workflow.

### Data analysis

The processed kinematic data from both systems was analyzed using custom-written Python software. To ensure high quality for the criterion method (MB system), the quality of the inverse kinematic processing was assessed for each trial by inspecting marker errors. Trials with RMS errors exceeding 2 cm or a maximum error over 4 cm were excluded^41,42,43^.

The data of both systems were first filtered with a low-pass Butterworth filter (30 Hz)^14^. In a second step, the time-series data of the MB and the Open Cap system were time-synchronized using waveform synchronization with a peak angle alignment approach. This was done with the initialization movement. This movement consisted of a maximum knee extension while standing on the box at a hip angle of ~ 90°. The time-point of peak knee extension angle was used to time-align both systems, and the alignment was visually checked for each trial by the principal investigator. Data before this peak was discarded. Synchronized datasets were refined using force plate data to identify IC and LO events (threshold: 20 N). Kinematic, kinetic, and GRF data were trimmed to the region of interest (ROI) from IC to LO, interpolated to 1001 samples, and time-normalized from 0% (IC) to 100% (LO) using piecewise cubic Hermite interpolation. The mean waveform for each parameter was calculated from ten valid trials per participant, and the overall mean ± SD was computed across all participants. Parameters included frontal knee kinematics, sagittal knee, and transverse hip joint kinetics, as well as vertical ground reaction forces^28^.

### Statistical analysis

For kinematic parameters, root mean square error (RMSE) was calculated on a participant’s individual basis across both systems and finally averaged across participants. The same was done for the maximum error for each analyzed parameter, representing the largest absolute difference between the two systems^21^. For joint moment and GRF data, the overall mean, SD, and error measures were calculated similarly to kinematic data. Instead of RMSE, the mean absolute error (MAE) was used^14^. MAE values for joint moments were normalized to the product of each participant’s body weight (BW) and height, whereas GRF data were normalized to each participant’s BW^14^. Maximum errors for joint moments and GRFs were calculated similarly to kinematic data. To identify statistically significant differences between the MB system and OpenCap, a statistical parametric mapping (SPM) analysis was conducted for the kinematic, joint moment, and GRF datasets using the SPM1D package (version 0.4.23) for Python^45^. A K^2^ test was used to determine if data was normally distributed. A paired sample, two-tailed t-test, was used for normally distributed data, whereas its non-parametric version was used for non-normally distributed data. The alpha level was set to 0.05.

Pearson correlation coefficients (r) were calculated to provide an easy-to-interpret measure of general inter-system waveform agreement for entire waveforms for kinematic, kinetic, and GRF data. Values of < 0.10 were interpreted as trivial, 0.10–0.39 as weak, 0.40–0.69 as moderate, 0.70–0.89 as strong, and > 0.90 as very strong correlations^23,46^.

A Bland-Altman plot^47^ was used to visualize inter-system agreements to evaluate OpenCap’s performance in determining discrete parameters relevant to ACLR re-injury risk, namely the sagittal knee joint moment asymmetry at IC^29^.

## Results

All 240 trials were eligible for kinematic analyses as no trial exceeded marker error thresholds. However, only 192 trials were included in kinetic analyses because 48 trials (20%) lacked optimal solutions from OpenCap’s dynamic simulations.

**Fig. 3:**
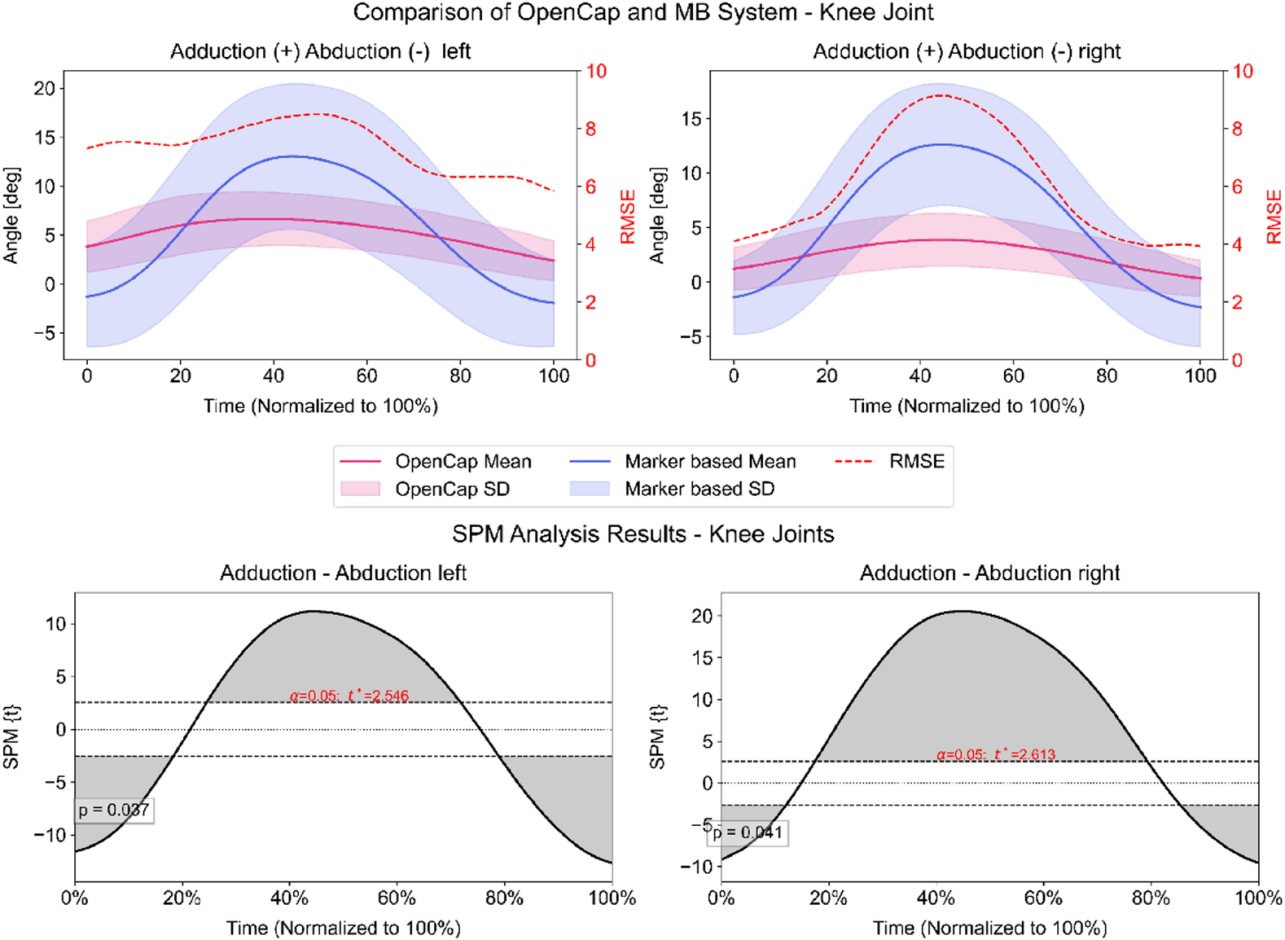
Upper plots: Frontal plane kinematics of both knee joints measured by the markerless (pink) and marker-based (blue) system. The secondary y-axis shows the mean root mean square error (RMSE) calculated per frame between both systems. Lower plots: Statistical parametric mapping results (SPM). Grey-shaded areas = statistically significant differences between the systems from initial contact to lift-off (100%). Dashed horizontal lines indicate the critical threshold (t*) for statistical significance (α = 0.05).

For frontal plane knee kinematics, an RMSE of 6.1 ° (± 1.9 SD) on the right and 7.4 ° (± 0.8 SD) on the left side were found between the methods. Maximum errors found were 12.2 ° (± 4.8 SD) on the right side and 14.5 ° (± 4.5 SD) on the left side. Waveform correlations between the methods were very strong on both sides (right: *r* = 0.97, left: *r* = 0.91, *p* < 0.001). However, SPM revealed statistically significant differences in ROI on the right side, ranging from 0 to 12%, 17–78%, and 84–100%, and on the left side, ranging from 0 to 18%, 24–71%, and 78–100% (Fig. 3).

**Fig. 4:**
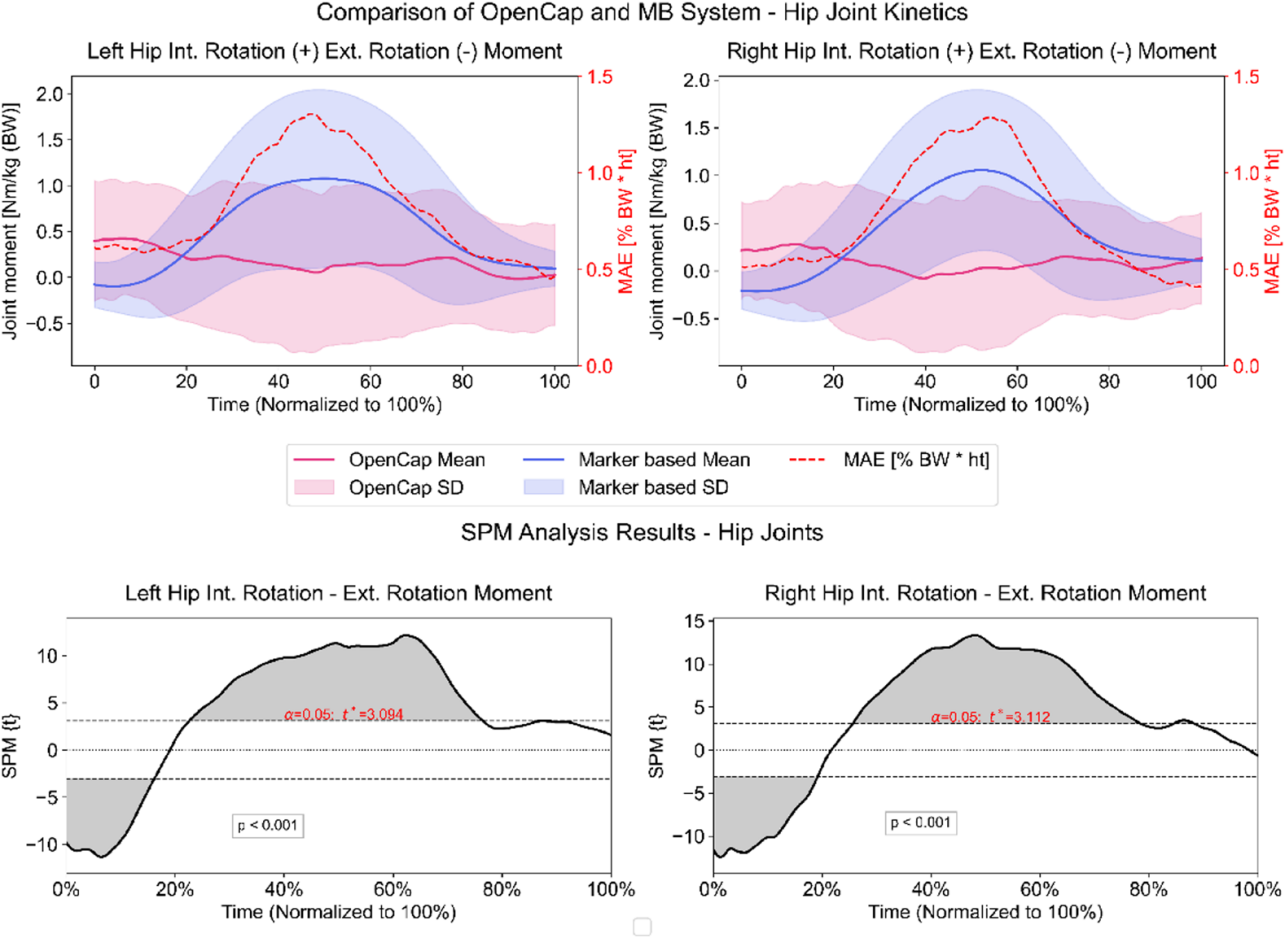
Upper plots: Transverse plane kinetics of both hip joints measured by the markerless (pink) and marker-based (blue) system. The secondary y-axis shows the mean absolute error (MAE) calculated per frame between both systems. Lower plots: Statistical parametric mapping results (SPM). Grey-shaded areas = statistically significant differences between the systems from initial contact to lift-off (100%). Dashed horizontal lines indicate the critical threshold (t*) for statistical significance (α = 0.05).

For kinetic outcome variables, an average MAE of 0.8% (± 0.3 SD) and a maximum error of 2.1% (± 0.8 SD) for transverse plane hip joint moments on both sides was present between the methods. Inter-system correlations were strongly inverse for the right side (*r*=−0.79, *p* < 0.001) and weakly inverse for the left side (*r*=−0.32, *p* < 0.001). Except for the first 20% of the ROI, OpenCap’s dynamic simulations underestimated hip rotation moments. Hence, SPM revealed statistically significant differences from 0 to 17%, 24–78%, and 84–88% on the right side and from 0 to 15%, 24–78%, and 81–92% of the ROI on the left side (Fig. 4).

**Fig. 5:**
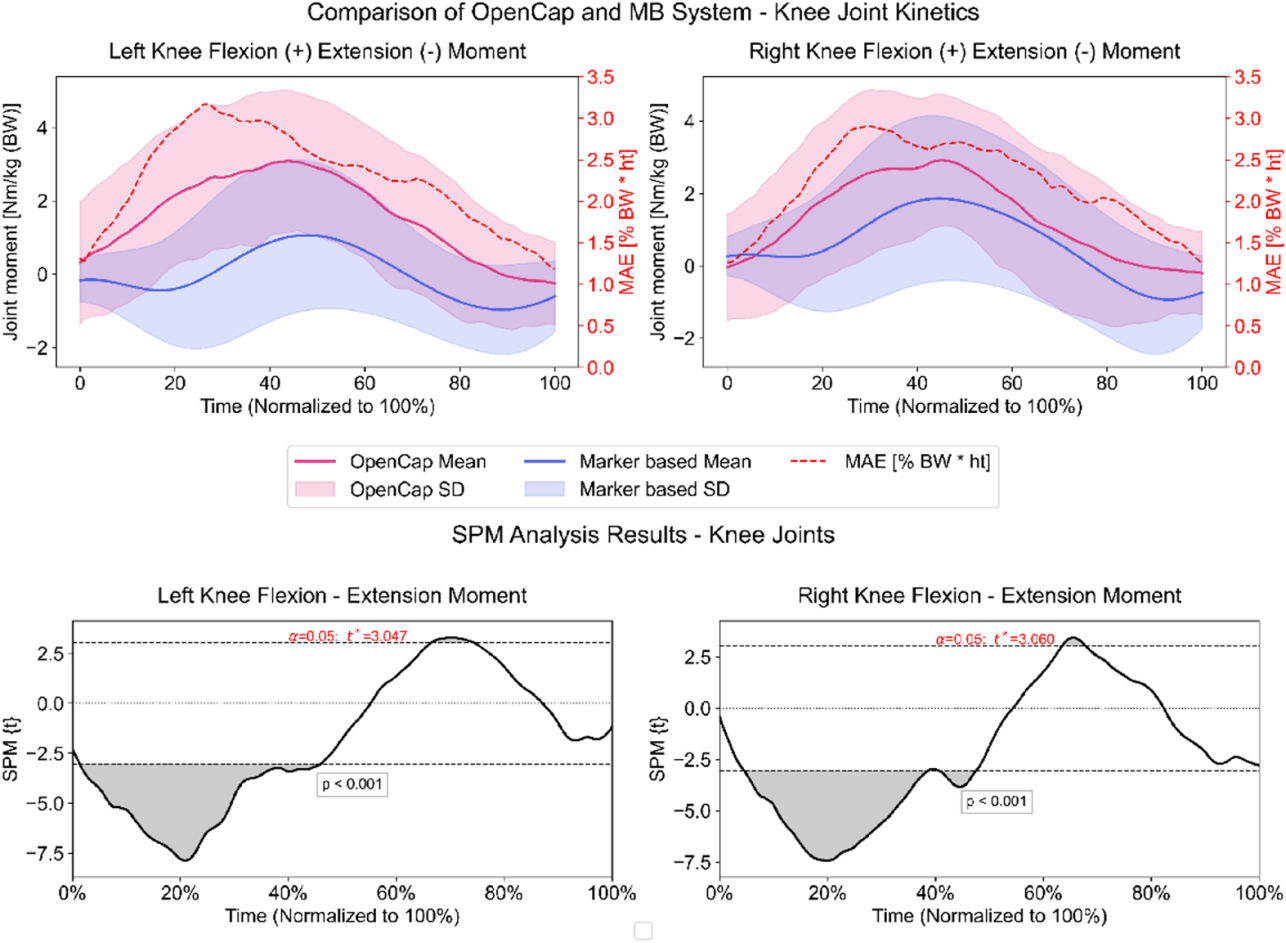
Upper plots: Sagittal plane kinetics of both knee joints measured by the markerless (pink) and marker-based (blue) system. The secondary y-axis shows the mean absolute error (MAE) calculated per frame between both systems. Lower plots: Statistical parametric mapping results (SPM). Grey-shaded areas = statistically significant differences between the systems from initial contact to lift-off (100%). Dashed horizontal lines indicate the critical threshold (t*) for statistical significance (α = 0.05).

Sagittal plane knee joint moments had a normalized MAE of 5.6% (± 1.7 SD) on both sides between methods. Average maximum errors reached 16.6% (± 6.1 SD) and 15.8% (± 6.1 SD) on the participants’ right and left side, respectively. The inter-system correlations for both sides were very strong (right: *r* = 0.92, left: *r* = 0.93, *p* < 0.001). SPM analyses, however, revealed statistically significant differences from 3 to 46% and 62–70% on the right side and from 2 to 44% and 65–76% of the ROI on the left side caused by OpenCap overestimating sagittal knee joint moments (Fig. 5).

**Fig. 6:**
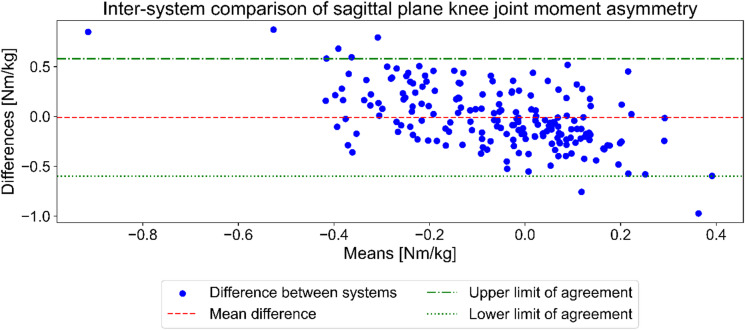
Bland-Altman plot of inter-system agreement in knee extensor moment asymmetry between OpenCap and the marker-based system. The Green dashed lines represent the limits of agreement (+/- 1.96 SD), and the red dashed line represents the bias between the methods. Note: The Y-axis represents differences between the systems calculated as OpenCap – Marker-based.

Concerning the side-to-side differences in knee extensor moment asymmetry at the instance of IC, the Bland-Altmann plot showed minimal bias between the systems of −0.0093 Nm/kg and limits of agreement of 0.6 Nm/kg for the upper limit and − 0.6 Nm/kg for the lower limit (Fig. 6).

**Fig. 7:**
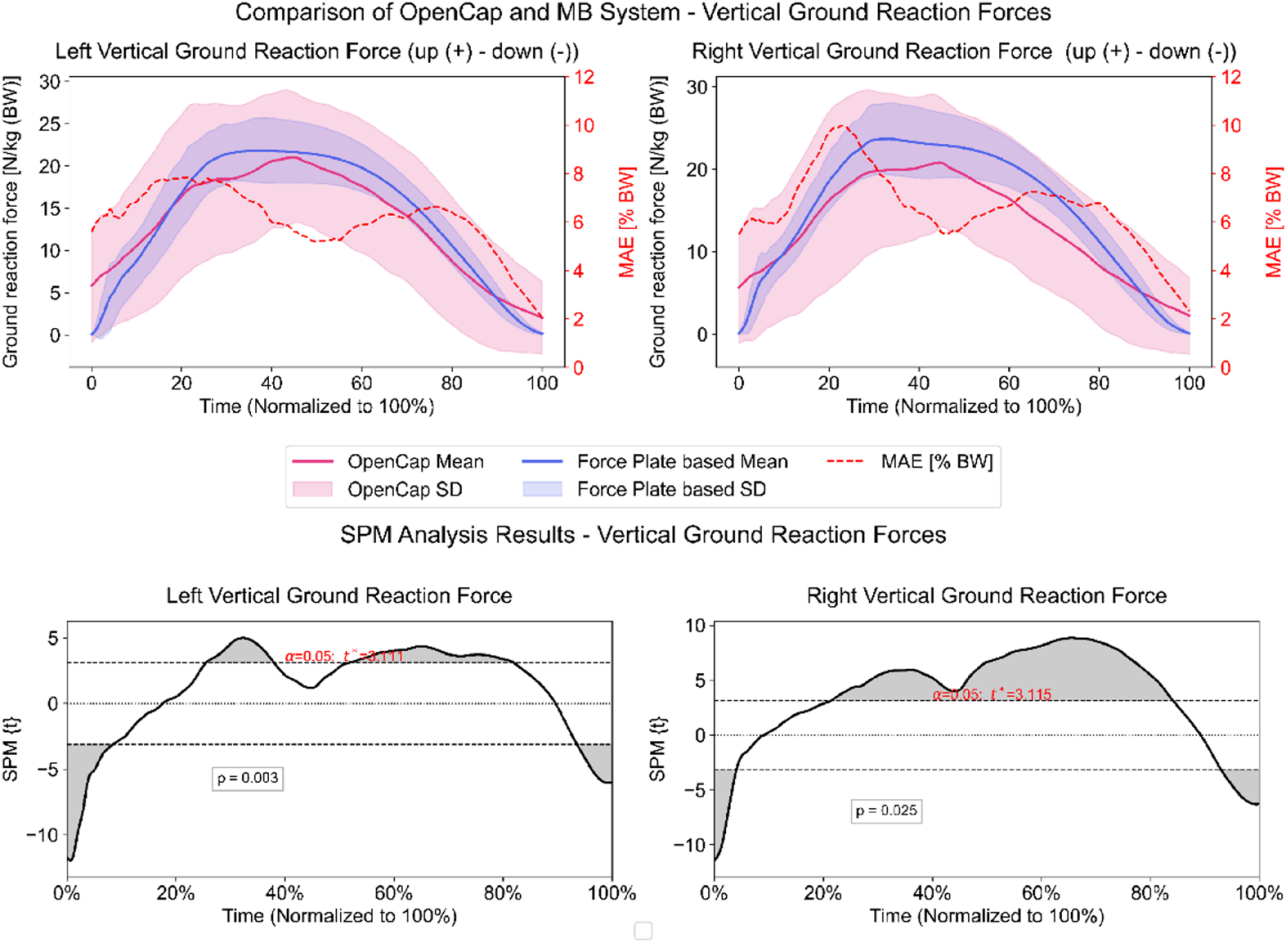
Vertical ground reaction forces of both sides measured by the markerless (pink) and marker-based (blue) system. The secondary y-axis shows the mean absolute error (MAE) calculated per frame between both systems. Lower plots: Statistical parametric mapping results (SPM). Grey-shaded areas = statistically significant differences between the systems from initial contact to lift-off (100%). Dashed horizontal lines indicate the critical threshold (t*) for statistical significance (α = 0.05).

The estimated vertical GRF showed very strong correlations to the force plate data on both sides (right: *r* = 0.97, left: *r* = 0.98, *p* < 0.001). The analysis showed a normalized MAE of 6.6% (± 1.6 SD) for vertical GRFs on the right side and 6.1% (± 1.3 SD) on the left side. Maximum errors reached 18.4% (± 6.3 SD) and 17.5% (± 6.5 SD) for the right and left sides, respectively. Statistically significant differences from 0 to 8%, 27–38%, 53–83%, and 94–100% of the ROI on the right side and from 0 to 4%, 24–88%, and 93–100% of the ROI on the left side were found via SPM (Fig. 7).

## Discussion

This study analyzed the concurrent validity of OpenCap compared to a traditional MB approach regarding kinematic and kinetic parameters related to an increased risk of ACL re-injury according to published literature^26,28,30^. To the best of the authors’ knowledge, this was the first independent study to simultaneously evaluate kinematic, joint moment, and GRF data derived using OpenCap in the context of an ACL re-injury screening using drop jumps.

From a kinematics perspective, frontal plane knee joint movement is often a focus when evaluating the risk of an ACL re-injury. Just recently, Templin et al.^48^ compared a drop vertical jump with a markerless (ENABLE system) and marker-based approach, showing an RMSE of 4.8° and an r of 0.61 for the frontal knee plane. Considering the shorter ground contact time and thus increased dynamics of the drop jump compared to the drop vertical jump, our results are comparable in terms of RMSE (~ 6°–7°) but superior with respect to the overall waveform agreement (*r* > 0.9) on the respective leg. However, Paterno et al.^26^ reported that a reduced frontal plane movement of 4.1° (second ACL: 16.2° vs. Healthy: 12.1°) during a drop vertical jump distinguished between patients who sustained a second ACL injury and those who did not. This value is in line with the reported minimum detectable change of 2.5°–5° for knee joint abduction obtained during treadmill running^24,25^. Although treadmill running involves lower dynamic demands than a drop jump, making it a less than perfect comparison, it still provides a meaningful reference point for interpreting our results. The larger RMSE and average maximum errors exceeding 12° suggest that OpenCap is not sufficiently accurate for kinematic evaluations related to ACL re-injury risk screening. This is further supported by our results of the SPM1D analysis, showing differences across almost the entire ground contact phase (Fig. 3). However, this consistent pattern of over- and underestimation in frontal plane knee kinematics during the contact phase (Fig. 3) may also indicate a systematic bias in the pose estimation algorithm. Since both datasets were processed using the same musculoskeletal model, these differences are likely due to the pose estimation step rather than discrepancies in the used model. Although this is speculative, it suggests that the observed error pattern reflects a systematic bias in the pose estimation algorithm, especially under high-impact conditions where rapid joint movements can challenge tracking accuracy. These findings highlight a potential area for algorithmic refinement to improve frontal plane motion tracking during dynamic tasks. Positively, waveform correlations between OpenCap and the marker-based system were very strong, indicating that the fundamental movement patterns were captured accurately. This suggests that future OpenCap versions could reduce error margins with further development and optimization to reach clinical relevance concerning evaluating ACL re-injury risk in the frontal knee plane. The reader should consider that our analysis was conducted using a modified and, so far, not natively supported version of OpenCap’s MSK model, which may have influenced our results.

Regarding kinetic parameters related to increased ACL re-injury risk^29^, sagittal plane knee joint moments showed promisingly strong waveform correlations between both systems (*r* > 0.9). However, normalized MAE of ~ 5.6% and normalized maximum errors > 15% exceeded the acceptable accuracy threshold of 2.5% for joint moments reported in literature^27^ and are slightly higher than data published by OpenCap’s developers on drop jumps^14^, (3.1 MAE, Supplementary S4 Table^49^. Additionally, SPM analyses revealed statistically significant differences for large parts of the ROI on both sides (Fig. 5). Because different MSK models (1 DoF vs. 2 DoF) were used, part of the discrepancy likely reflects methodological rather than performance differences (see Limitations). In contrast to sagittal plane knee moments, differences found for hip rotation moments between OpenCap and the MB system were within the desired error threshold of 2.5%^44^ and within the range reported by Uhlrich et al.^14^, 1.3%, Supplementary S4 Table^49^. However, correlation analysis showed negative correlation coefficients, meaning that the general pattern of the two methods was different. A possible explanation for the negative correlation between OpenCap and the MB approach may be attributed to the estimated shear components of OpenCap (Figure.S1). It is obvious that the estimated medio-lateral and the anterior-posterior forces exhibited higher variability. This increased variability likely contributes to inaccuracies in estimating the center of pressure (COP), ultimately influencing the calculated hip joint rotation moments. Moreover, OpenCap relies on joint center locations derived from OpenPose, a markerless algorithm known to exhibit systematic deviations from marker-based estimates due to potential labeling inaccuracies in its training data^50^. These factors collectively may explain the discrepancies observed in hip rotation moment estimation between the two methods. The SPM analysis further supports this, showing differences in large portions of the region of interest (Fig. 4). Overall, despite strong waveform correlations, the errors in sagittal plane knee joint moments indicate that OpenCap is not yet precise enough for kinetic evaluations related to ACL re-injury risk. Although hip rotation moments were within desired error margins, the negative correlations and, hence, differing movement patterns highlight inconsistencies that limit its current clinical applicability.

Knee extensor moment asymmetry at IC was analyzed using a Bland-Altman plot (Fig. 6). Although the low bias between OpenCap and the MB system indicated high accuracy, the wide limits of agreement (−0.6 to 0.6 Nm/kg) suggested poor precision. These limits exceed the previously reported differences of approximately 0.1 Nm/kg in knee extensor asymmetry at IC among participants who suffered an ACL re-injury^26^. This suggests OpenCap is not yet accurate enough to replace established criterion methods for measuring joint moments during a drop jump at IC.

Lowered vertical GRFs are often seen in the affected limb in ACL patients, as shown by literature on male and female athletes 9-months post-surgery, and are discussed as a learned compensation pattern^28,30^. Vertical GRFs on both sides showed very strong waveform agreements (*r* > 0.97), again demonstrating OpenCap’s ability to capture the fundamental pattern of the waveform. With an MAE of ~ 6.5%, results were promising and surprisingly lower than the original publication by Uhlrich et al., reporting an MAE of 25.2% for the vertical GRF of a Drop Jump^14^ (Supplementary S3 Table^51^). A possible reason for the improved results in this study might be the increased sample rate (240 Hz) compared to the 60 Hz used by Uhlrich et al.^14^, resulting in an improved temporal resolution of the high-frequency content of GRF profiles. However, our results indicated a maximum error of 17–18%, which limits clinical application, particularly at the individual level. Our SPM analysis further supports this, which revealed statistically significant differences on both sides of the body across large sections of the ground contact phase (Fig. 7).

The study has several key limitations. The results shown here apply only to OpenCap’s marker augmenter v0.3. Future versions may produce different results. Only healthy participants were included, limiting generalizability to ACLR-affected populations. A notable limitation of this study was the exclusion of 20% of the kinetic trials due to the absence of optimal solutions during OpenCap’s dynamic simulations. Ground reaction forces were modelled using six contact spheres under each foot^52^, which are then combined with kinematic inputs to compute joint moments. This process is sensitive to noisy data and may fail when the optimization does not converge. No adjustments were made to OpenCap’s default simulation settings to reflect a less experienced end-user approach. Although targeted tuning of simulation parameters (e.g., cost function weights) could likely reduce the failure rate, such adjustments require advanced biomechanical expertise. Therefore, this limitation reflects a realistic use-case scenario for non-expert end-users. Waveform synchronization between the marker-based system and OpenCap was achieved using a peak alignment approach, which had shown the most consistent results during internal testing. Although no quantitative peak alignment error data were systematically collected, alignment for each trial was visually verified to ensure sufficient temporal matching of the main kinematic events. However, any misalignment may have affected our findings, resulting in increased RMSE values. A further limitation is that different MSK models were used for kinetic analyses across systems. Kinetic analysis in OpenCap requires the native model with a 1-DOF knee joint because the 2-DOF knee model used for kinematics is currently not supported for kinetic simulation. Hence, OpenCap and MB kinetics were generated from different knee models, introducing a potential source of inconsistency and likely contributing to the higher errors observed in knee joint moments. In addition, the lack of support for 2-DOF knee kinetics restricts the analysis of frontal-plane knee loads and presently limits OpenCap’s ability to reproduce ACL-relevant metrics attainable in a motion analysis laboratory.

## Conclusion

OpenCap demonstrated strong waveform correlations with a traditional marker-based approach when assessing kinematics, joint moments, and ground reaction forces during drop jumps. However, clinically relevant errors - especially in the frontal plane knee angle, knee joint moments, and knee extensor moment asymmetry at initial contact - exceeded thresholds reported in the literature for detecting ACL re-injury risk. While OpenCap showed promise in capturing overall movement patterns, further refinement is needed before clinical application, including improvements to kinetic simulations, which did not complete successfully in approximately 20% of trials. Considering the rapid developments in pose estimation and GRF estimation using data-driven approaches, future efforts to refine OpenCap should first focus on reducing kinematic errors through improved pose estimation methods. Once more robust kinematic estimates can be achieved, research should focus on enhancing musculoskeletal modeling and simulation algorithms to strengthen kinetic estimates and clinical utility.

## Supplementary Information

Below is the link to the electronic supplementary material.

Supplementary Material 1

## Data Availability

The datasets used for the current study are available from the corresponding author on reasonable request.
