# Supplementary material for: Concurrent validation of OpenCap for identifying ACL re-injury risk factors during a drop jump test in a healthy cohort
Source: Sci Rep. 2026 Mar 24;16:9843. doi: 10.1038/s41598-026-44758-0 (PMC13018474; doi:10.1038/s41598-026-44758-0)
Supplement: Supplementary file 1 — Supplementary Material 1 [file 41598_2026_44758_MOESM1_ESM.pdf]

Figure.S1:

The figure below shows the analysis of the three components of the ground reaction force for both the left and right sides. The figure clearly shows the increased variability in the anterior–posterior and medial–lateral forces estimated by OpenCap compared to the force plate data used in the MB approach. Additionally, SPM analysis performed on these data reveals significant differences between OpenCap and the MB device (highlighted by the grey shaded area). Please refer to the Discussion section for further details. Note: To obtain a comprehensive view, the vertical ground reaction force as shown in the manuscript is also presented.

Comparison of 3D ground reaction forces between systems

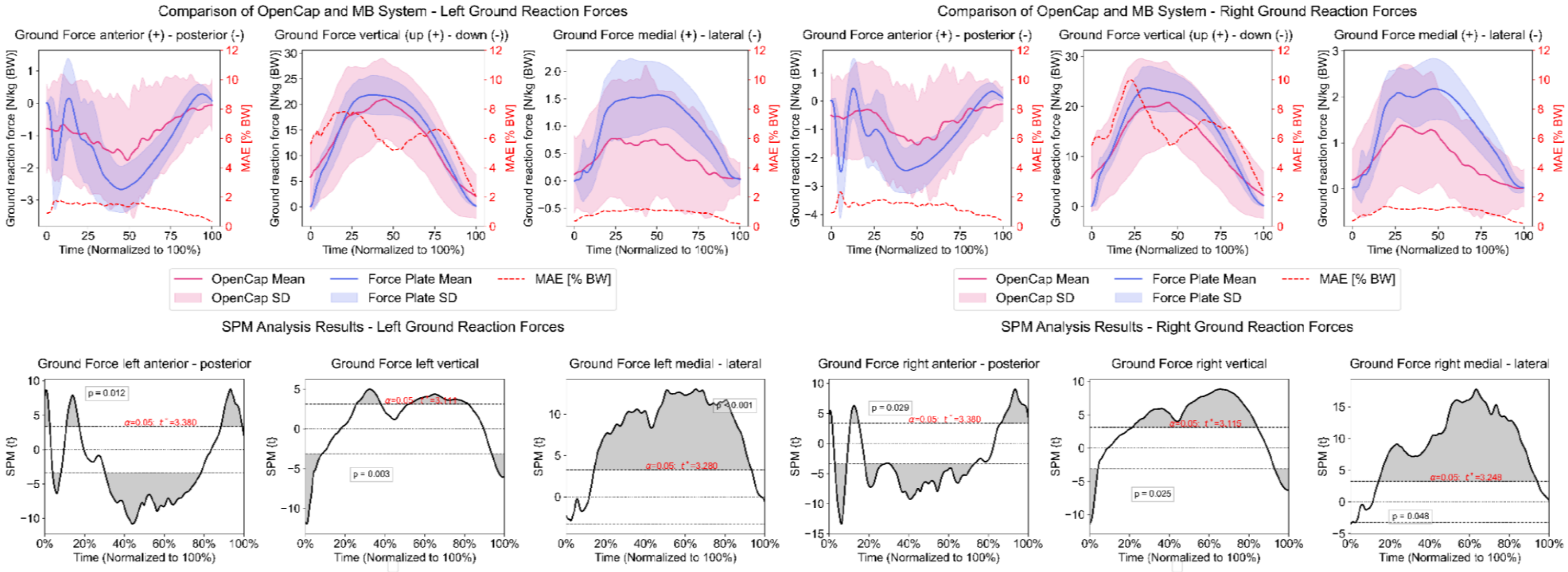

Figure.S1: First row: Comparison of anterior-posterior, vertical, and medial-lateral ground reaction forces of both sides measured by the markerless (pink) and marker-based (blue) system. The secondary y-axis shows the mean absolute error (MAE) calculated per frame between the two systems. Second row: Statistical parametric mapping results (SPM). Grey-shaded areas = statistically significant differences between the systems from initial contact to lift-off (100%). Dashed horizontal lines indicate the critical threshold (t) for statistical significance (α=0.05).*
